# Is Newborn Screening the Ultimate Strategy to Reduce Diagnostic Delays in Pompe Disease? The Parent and Patient Perspective

**DOI:** 10.3390/ijns6010001

**Published:** 2020-01-09

**Authors:** Raymond Saich, Renee Brown, Maddy Collicoat, Catherine Jenner, Jenna Primmer, Beverley Clancy, Tarryn Holland, Steven Krinks

**Affiliations:** Australian Pompe Association Inc., Kellyville, NSW 2155, Australia

**Keywords:** Pompe disease, newborn screening, diagnosis, infantile onset Pompe disease, late onset Pompe disease, patient perspective

## Abstract

Pompe disease (PD) is a rare, autosomal-recessively inherited deficiency in the enzyme acid α-glucosidase. It is a spectrum disorder; age at symptom onset and rate of deterioration can vary considerably. In affected infants prognosis is poor, such that without treatment most infants die within the first year of life. To lose a baby in their first year of life to a rare disease causes much regret, guilt, and loneliness to parents, family, and friends. To lose a baby needlessly when there is an effective treatment amplifies this sadness. With so little experience of rare disease in the community, once a baby transfers to their home they are subject to a very uncertain and unyielding diagnostic journey while their symptomology progresses and their health deteriorates. With a rare disease like PD, the best opportunity to diagnose a baby is at birth. PD is not yet included in the current newborn screening (NBS) panel in Australia. Should it be? In late 2018 the Australian Pompe Association applied to the Australian Standing committee on Newborn Screening to have PD included. The application was not upheld. Here we provide an overview of the rationale for NBS, drawing on the scientific literature and perspectives from The Australian Pompe Association, its patients and their families. In doing so, we hope to bring a new voice to this very important debate.

## 1. Introduction

Pompe disease (PD), also known as glycogen storage disease II or acid maltase deficiency, is a rare, progressively debilitating lysosomal storage disorder. It is named after Joannes Cassianus Pompe, who first described a case of idiopathic hypertrophy of the heart in a 7-month old infant in the Netherlands in 1932, noting massive vacuolar glycogen accumulation not only in the heart but in all tissues examined [1].

Affected patients have an autosomal-recessively inherited deficiency in the enzyme acid α-glucosidase (GAA, also called acid maltase). This deficiency leads to accumulation of glycogen in multiple tissues, especially in the skeletal muscles, heart, and liver [2]. These glycogen deposits disrupt muscle cell architecture and function, causing progressive motor, respiratory, and cardiac dysfunction. While both genders are equally affected, PD is a spectrum disorder in which the age at symptom onset and rate of deterioration can vary considerably.

The clinical impact of PD is determined primarily by the amount of residual GAA enzyme activity. Enzyme activity is absent or minimal (<3%) in infantile-onset disease (IOPD), but may be reduced to varying degrees (3%–30%) in those with juvenile-onset (JOPD) or late-onset disease (LOPD) [3]. A lower residual enzyme activity level is associated with earlier onset, more severe disease, faster progression, worse prognosis and a shorter survival time [4]. In IOPD clinical symptoms typically become apparent within the first few months of life; prognosis is poor such that without treatment most infants die within the first year of life [5].

The rarity of PD combined with a variety of overlapping clinical signs and symptoms hamper its diagnosis and the initiation of therapy [6,7,8]. Such delays have a significant negative impact on patients and their families. Recent research has shown that newborn screening (NBS) appears to be better at identifying PD cases than does clinical examination, especially for classical IOPD [9]. Is NBS the ultimate strategy to reduce diagnostic delays in PD? Here we provide an overview of the rationale for NBS, drawing on the scientific literature and including perspectives from patients and their families. In doing so, we hope to bring a new voice to this very important debate.

## 2. Diagnostic Delay

Diagnostic delay is common in PD, and it exists across the disease spectrum [8]. Data from the Pompe Registry has found the diagnostic gap to be shortest (average 1.4 months; range: 0.0–13.9 months) in patients with classic IOPD and longest in patients with JOPD (average 12.6 years; range: 0.0–60.0 years). Delays are also significant for LOPD (average 6.0 years; range: 0.0–49.8 years) [8]. Within Australia, IOPD diagnosis can occur within a few months of initial symptom onset, but diagnostic delays of up to 7 months have been reported in the literature [10].

The impact of this delay is such that for many patients, health and functional status is often already severely impaired at the time of their diagnosis. Analysis of data from 53 patients (age range 0–64 years) has shown that at the time of diagnosis [11]:

- Classic IOPD patients—cardiac function, hearing, muscle strength and motor development were all impaired, one in three (36%) required supplemental oxygen and two in three (64%) required nasogastric tube feeding;
- LOPD patients—advanced muscle weakness and impaired respiratory function were present, causing varying degrees of handicap, and respiratory support (14% of adults) and use of a wheelchair (7% of adults) were required.

### 2.1. Barriers to Timely Diagnosis—Australian Perspectives

Although not specific to PD, prompt diagnosis of rare diseases can have many important positive ramifications. Prompt diagnosis facilitates access to appropriate treatment, it can help parents to better understand their child’s condition and explain it to others, it may reduce the burden of blame parents feel and it may alleviate some of the stress of the unknown [12].

Australian research (Table 1) has highlighted a lack of screening tests and limited knowledge amongst healthcare professionals as key barriers to diagnostic delays in rare diseases, with the authors calling for more educational support and wider access to a multi-disciplinary team approach to patient care [13]. Australian pediatric research has shown that more than half of the children with a rare disease were not diagnosed until after referral to a clinical specialist in a large metropolitan pediatric hospital [12], confounding the diagnostic delay.

Specialist referral is reported as a pivotal step in obtaining a clinical diagnosis of rare diseases in Australia [12]. In accordance with this, a recent European survey exploring diagnostic odyssey in PD found that circuitous involvement of several healthcare professionals increased the diagnostic delay of IOPD by 200% compared to direct referral to a specialist center [14].

Australians living in rural and remote areas have additional diagnostic barriers, confounded by lack of proximity and timely access to such specialist healthcare providers. In such cases, the typical care pathway involves multiple steps—initial parental recognition that something is not right with their baby, general practitioner (GP) acknowledgement, and referral to a pediatrician—before the child can be seen in a specialist center. This stepwise approach can take a minimum of 3 months and can place a significant financial and psychosocial burden on the family. Some babies survive the time it takes to confirm a diagnosis, but many do not.

### 2.2. Diagnostic Delays—Australian IOPD Experiences

In the case of PD, the presenting symptoms are diverse and not likely to be suggestive of this diagnosis unless another family member or relative has already been diagnosed [15]. A high index of clinical suspicion is needed to ensure that patients are appropriately examined and tested. A doctor with intimate experience in the diagnosis or management of PD may be more likely to recognize symptoms in an undiagnosed patient; unfortunately very few doctors will have seen a PD patient in Australia. Testing for PD is a relatively simple procedure that can be undertaken in a few days. Clinician awareness of, and increased vigilance for, the early symptoms of PD are therefore also important contributory factors to timely diagnosis [10].

**Figure 1:**
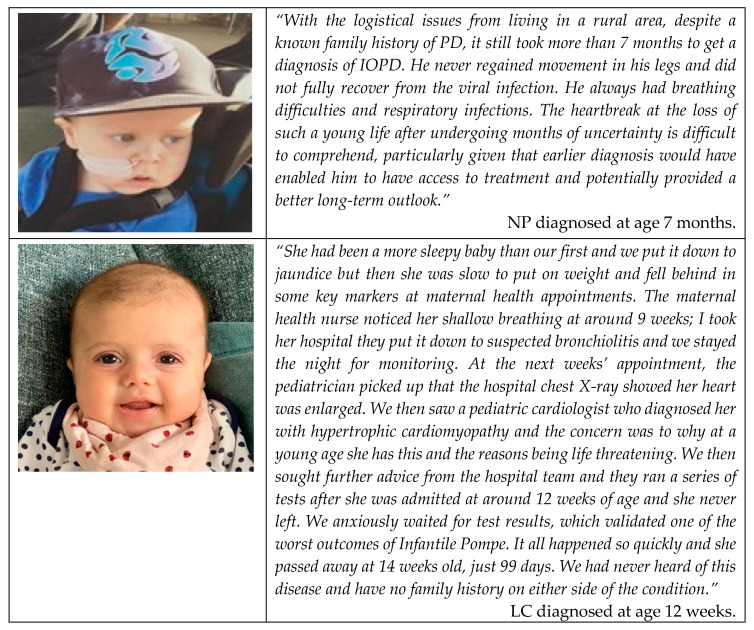
Parents’ perspectives on Australian infantile-onset disease (IOPD) diagnostic experiences. In lieu of patient informed consent, photographs and comments have been provided by, and reproduced with permission from, the parents of these two children both of whom were deceased at the time the paper was written.

A particular case in point is that of NP (Figure 1). NP was born 4–6 weeks prematurely; whilst he developed normally at first, early symptomology became apparent within the first few months of his life. Hypotonia, dysphagia, and developmental delays resulted in the family GP diagnosing failure to thrive at age 2 months and NP was placed on a wait-list for pediatrician referral. At age 6 months, not having seen the pediatrician, NP contracted a viral infection. He was hospitalized initially in a regional hospital 58 km from home and transferred 2 days later to the main tertiary hospital, 168 km away. Upon being admitted to the tertiary hospital blood samples were taken and sent interstate (to South Australia) and overseas (to the USA) for analysis. Ten days later the results were returned and NP was formally diagnosed with IOPD. The extent of physical damage sustained to his body during the time to diagnosis was such that NP was unable to survive, passing away aged 32 months.

In the last 5 years, the Australian Pompe Association is aware of at least four infants passing away in their first few months of life. These include a baby girl (LC, Figure 1), who was diagnosed at age 12 weeks and three baby boys the youngest of whom died at age 15 weeks. A further two young children, one of whom was diagnosed at age 3 months and died at 19 months and the other who was diagnosed at 9 months and died at 24 months, both had commenced enzyme replacement therapy (ERT) but experienced substantial immune responses [10].

## 3. What Do We Know about NBS for PD?

The overarching aim of any NBS program is to improve early identification of patients with treatable genetic metabolic disorders diseases in order to confirm diagnosis and initiate management to improve overall health outcomes [16]. Since the initiation of NBS for phenylketonuria, the criteria published by Wilson and Junger in 1968 have provided guiding principles to determine which conditions should be added to the panel [17]. These criteria include availability of a suitable screening test, an effective treatment, an early onset form of the disease that would be debilitating if not treated soon after birth, and consideration of overall cost-effectiveness [18].

Population NBS for PD was first implemented in Taiwan in 2005. A decade later, in 2015, it was added to the USA Department of Health and Human Services-endorsed Recommended Uniform Screening Panel (RUSP) [19]. As of November 2019, implementation has occurred in 23 states and a further 9 states are actively pursuing implementation or conducted pilot studies [20]. Including Taiwan, an estimated 239,333,512 people benefit from the protection of NBS for PD. Pilot NBS studies have also been conducted in Austria, Italy, Hungary, and Japan [21]. Whilst establishment of these programs is challenging and controversial, key driving factors include [19]:

- The development of promising new treatment options;
- Advances in screening technology;
- Advocacy by special interest groups.

### 3.1. Benefits of NBS for PD

#### 3.1.1. Reduced Diagnostic Odyssey in IOPD

The primary benefit of NBS for PD is the potential to identify patients before symptoms arise, enabling timely initiation of therapy before irreversible damage occurs. Babies with IOPD identified via NBS would be eligible to receive treatment at around 22 days of life, compared to 4 or 5 months of age when relying on symptom-based referral and subsequent diagnosis [8].

Research based on using different decision-analytic models has demonstrated NBS to be superior to clinical examination in identifying IOPD cases, with a significant, positive impact on projected health outcomes [9]. Identifying 40 cases of IOPD via NBS would avert 13 (range 8–19) deaths and 26 (range 20–28) cases of ventilator dependence amongst babies surviving to 36 months of age, assuming all children were treated with ERT. The authors compared their analysis to available real-world data from infants who had undergone NBS for PD in the USA pilot studies at that time, noting that this data supported their models and indicated that the number of cases likely to be detected would be at the upper range of these predictions [9].

#### 3.1.2. Greater Knowledge of Reproductive Risk

At present PD is carried undetected in the community. While patients who have a family history of PD are cognizant of its impact, for many patients there is no such history and a diagnosis of PD can devastate these unexpecting families. NBS provides secondary benefits that positively impact these families, and the wider community, by creating an opportunity to inform about reproductive risks [22]. Early diagnosis of LOPD enables these patients to make better-informed choices regarding future family planning. In addition, a positive NBS screen can be the stepping-stone to identifying reproductive risks in the parents before the birth of a second child. NBS will provide recourse to advice and options, and with knowledge comes informed choices.

Clear frameworks would need to be established to ensure patients and their families are closely followed-up and provided with access to genetic counseling. Equipping key healthcare providers, such as the GP, with information about carrier results and their reproductive implications may help to facilitate parents’ understanding of their child’s NBS results.

#### 3.1.3. Improved Understanding of the True Prevalence of PD

PD has been thought of as a rare genetic metabolic disease; estimates vary but the literature generally states an incidence of approximately 1 in 40,000 births, of which one-third are infants with IOPD. The Australian Pompe Association membership register does not reflect these IOPD numbers. The youngest treated child currently listed in the Australian Pompe Association membership register is 5 years old; the group is aware of a younger child, aged 4 years, who is diagnosed but not a currently registered member of the Association.

Published Australian data from the mid-1990s estimated an incidence of 1 in 201,000, a prevalence of 1 in 146,000 and a carrier frequency of 1 in 191,000 [23]. No breakdown on the type of PD was provided. As part of reforms into the Government-funded Life Saving Drug Program, a protocol has been established to review the prevalence of PD in Australia based on literature and other published data sources [24]. Data from this review are not yet available, but it is recognized that it will be limited by the availability and incompleteness of identified datasets.

NBS for PD provides a means to help better quantify the true prevalence of this disease. The number of PD cases identified in NBS programs are summarized in Table 2 [21]. These data demonstrate that NBS is finding up to twice the number of cases than were previously thought to exist, underscoring the immense difficulty in diagnosing this condition based on clinical symptomology alone.

It is possible that in Australia alone up to two or more babies may die every year without ever having been diagnosed with their underlying IOPD; with these deaths being registered instead as cardiomyopathy or other symptoms of unknown cause. Importantly, the diverse origins of the Australian population may alter the prevalence of PD in the community, but a true understanding of this will not be known without including PD in the national NBS program or, at the very least, investing in a pilot NBS study.

### 3.2. What Have We Learnt from Current PD NBS Programs?

NBS for PD was first piloted in Taiwan in 2005 and introduced into the Taiwan NBS panel in 2007 [25]. Modification of methodology and systems over time has resulted in a substantial shortening of time to first diagnosis from 19 to 9 days and the time to first treatment initiation from 26 days to 1 day after IOPD confirmation.

Many factors can influence an individual’s response to treatment with ERT, including their age and the extent of preexisting pathology [26]. Experience in Taiwan shows that the sooner ERT is commenced, the better the results are [27]. Commencing ERT even a few days earlier can lead to better patient outcomes [28]. For example, 100% of the patients identified through NBS in Taiwan who were initiated on ERT at 6–34 days, and treated for a median of 63 months, remained ventilator-free and have been able to meet age-specific developmental milestones, such as normal independent walking age [29]. By comparison, studies assessing the long-term outcomes of IOPD patients in countries that do not have NBS (including the UK, Germany, the Netherlands, and Italy) report that a substantial number (27%–40%) of children pass away within the first years of life despite ERT [30].

The Taiwanese experience demonstrates that earlier intervention with ERT in IOPD cases can have a positive impact by reducing the future burden of this disease. Moreover, the Erasmus university PD variant database (http://www.pompevariantdatabase.nl/pompe_mutations_list.php?orderby=aMut_ID1) provides data linking the progression and outcomes of over 860 patients with their genetic errors. If genetic sequencing is included as part of NBS confirmatory protocols, this information provides an opportunity to better understand the mutation and to more clearly predict the outcome for the patient.

On this basis, we are of the opinion that the costs of early identification via NBS and early initiation of ERT treatment could be offset by the potential for improved patient quality of life, reduced disease-related disability (such as reduced need for ventilatory support) and reduced associated costs. However, we are unable to support this with hard data at present. Importantly, we remain cognizant that that the current treatment for PD is a first generation product. With considerable research underway for second generation ERT and the potential of gene therapy, the current challenge is to keep Pompe babies as well as possible with the technology available today until a cure is available tomorrow.

### 3.3. Impact on Immunomodulation Protocols

The development of high and sustained antibody titers (HSAT) is most often associated with IOPD patients who are cross-reactive immunological material negative (CRIM-negative) leading to the recommendation that these patients receive prophylactic immunomodulatory therapy [31]. Whilst ERT has improved clinical outcomes for many patients with PD, there is a risk of developing anti-drug antibodies. HSAT can be associated with worse clinical outcomes. Prophylactic and therapeutic immunomodulation reduce antibody levels, but questions remain as to optimal timing and protocols [32]. HSAT has also been observed in some CRIM-positive patients, raising questions as to how to determine which of these patients should also receive prophylactic immunomodulation [26].

Initiating ERT within the first month of life has not been shown to prevent HSAT [26]. However, experience from Japan, not undertaken in the context of a NBS setting, suggests that early initiation of ERT in the pre-symptomatic period may prevent the progression of IOPD and reduce the likelihood of anti-drug antibody production [33]. These authors suggested that starting ERT before the immune system had matured might have enabled natural immune tolerance. Further research is clearly needed in this area, but this finding opens up possibilities of additional benefits for NBS beyond diagnosis and treatment initiation, because earlier treatment may modify the need for and extent of immunomodulatory approaches.

### 3.4. Weighing Prognostic Uncertainty against Informed Decision Making

#### 3.4.1. False Positives

Identification of false positive and subsequent prognostic uncertainty is always going to be a core consideration with any NBS program. The extensive Taiwanese experience reports a false positive rate of 0.02% in IOPD targets and 0.01% in IOPD/LOPD targets. Similarly low false positive rates of 0.04% (38.3 per 100,000) and 0.05% (53.2 per 100,000) have been reported from pilot NBS programs in Missouri and Illinois [17] and in a Japanese feasibility study (false positives 0.3%, 2/530) [34].

The literature demonstrates that while prognostic uncertainty does cause heightened anxiety amongst parents in the short-term, there are no documented long-term harms [35]. Options are available to mitigate the issues surrounding identification of false-positives. For example, integrating tandem mass spectrometry with multivariate pattern recognition software to determine which patients warrant second-tier confirmatory testing has been evaluated in the USA with good results, significantly reducing the false-positive rate [36]. The investigators involved in the New York State pilot NBS program suggest the use of second-tier molecular analysis to reduce the burden of referral in screen-positive infants [37]. With their long history of experience in NBS for PD, Taiwanese experts also suggest a second-tier test to reduce the rate of false-positives and facilitate referral of true positives [38].

#### 3.4.2. Early Identification of LOPD

Given that PD is a spectrum disorder, NBS also has the potential for identifying babies with LOPD, noting that their clinical symptomology would not manifest until later in life. Current estimates of the Australian Pompe Association would suggest that if Australia first-tier testing of dry blood spot samples were to take place, for every IOPD case identified there would also be 6 LOPD cases identified. This brings with it several ethical questions surrounding when to start treatment and the burden placed on the patient and their family in terms of waiting for symptoms to appear [39,40].

In the Taiwanese NBS program, 473,738 newborns were screened and 19 LOPD cases had been identified by 2011; 6 (32%) of these patients had commenced ERT between the ages of 1.5–36 months [25]. All 6 patients showed abnormalities with glycogen storage prior to commencing treatment; currently, aged 8–13 years, they have met normal developmental milestones. The remaining 13 patients have not been treated and continue to develop normally, likely representing a milder phenotype [25]. This experience suggests that close monitoring of symptoms and timely ERT initiation, in combination with genetic counseling, education and support should form key aspects in the long-term care of less severe LOPD patients identified through NBS. The ultimate aim being to minimize early medicalization of children while at the same time providing robust protocols to assure the appropriate provision of available treatments.

Aside from treatment and care considerations, there are many positives that must also be considered in the early detection of LOPD cases. These include the ability to learn more about the natural history of the disease and to better As we enter the 2020s equip individuals to make informed choices later in life regarding family planning issues, as discussed above.

The humanistic burden of LOPD is high [41]. Misdiagnosis further impacts this, in terms of costs to the healthcare system incurred as a result of multiple tests and medications being tried without treating the underlying cause and to the patient in terms of ongoing uncertainty, dealing with symptoms and never being quite sure if they would have a better quality life now had they been diagnosed earlier. Early diagnosis of LOPD enables more timely management and may help prevent complications and improve outcomes now that therapy is available [42].

Consider a patient in their 30–40s with mild symptomology. The differential diagnosis for PD is so wide that confirmation of diagnosis may take several years (estimates are in the range of 5–8 years). Importantly anecdotal experience dictates that diagnosis frequently comes after a crisis or other major life event, such as after childbirth, creating additional complexity to what is already a potentially difficult situation.

Without NBS, LOPD patients may go for many years endeavoring to find a diagnosis. The Community loses so much through the cost of unnecessary health care visits while patients struggle for years to find a diagnosis for the want of a simple test at birth that would have alerted the parents or the patient. From a patient’s perspective a key advantage to early identification of LOPD via NBS is that it vastly reduces their personal diagnostic journey. In the words of an Australian PD patient, diagnosed in 2010 after a 13-year diagnostic journey:

*“There is much that needs to be done to help people with rare diseases, particularly around raising awareness to the public and also medical professionals in order for early diagnosis and also correcting misdiagnosis to occur. Had I been diagnosed even in 1997 when I was 17 and received treatment as soon as it became available perhaps my life would be very different today.”*

## 4. Specific Considerations for NBS in the Australian Setting

### 4.1. Current NBS Policies and Processes

Within Australia, NBS is offered free of charge, and, although not compulsory, participation is high [43]. Australian NBS programs began with screening for phenylketonuria (1967), followed by the addition of congenital hypothyroidism (1977), cystic fibrosis (1981 in New South Wales, 1999 in all other states) and galactosemia (early 1980s). In the late 1990s with advances in technology the list has expanded to include around 25 different disorders [44]. There had been a long silence, with no new conditions added for over 17 years, until the recent initiation of two pilot studies in New South Wales, for primary immune deficiency and spinal muscular atrophy.

All NBS services are coordinated from five centralized screening laboratories (one each in New South Wales, Queensland, South Australia, Victoria, and Western Australia). However, prior to 2018, specific program policies and which conditions to include were individually decided by each state jurisdiction. This has now been replaced with a National Policy Framework, which is accessible via the internet (http://www.cancerscreening.gov.au/internet/screening/publishing.nsf/Content/newborn-bloodspot-screening) [45]. This framework unites these programs for the first time since their inception over 50 years ago. Importantly, it provides a nationally agreed vision and way of working and outlines what will be needed to ensure the ongoing success of the NBS program in Australia; noting that state and territory governments will have the final responsibility for adding the condition in their jurisdictions.

As part of this framework, there is now a national evidence-based process to evaluate proposals to include new conditions in the NBS program [45]. This framework requires the provision of published evidence to support the condition proposed, the test that will be used and the availability and efficacy of treatment such that decisions to include new conditions can be made in line with agreed criteria. These criteria include consideration of (1) whether there is benefit to the baby from early diagnosis of conditions screened, (2) whether the benefit is reasonably balanced against any harms and costs, (3) the availability of a reliable test suitable for newborn bloodspot screening and (4) the availability of a satisfactory system in place to deal with diagnostic testing and follow-up care of babies with abnormal screening results.

The National Policy Framework sets out a step-wise decision-making pathway to carefully evaluate all applications [45]. Following a request to include a new condition in the NBS program and assessment of the available evidence, the possible outcomes include a recommendation to screen, a recommendation to conduct a pilot study, a recommendation to review at a later point in time and a recommendation not to screen.

### 4.2. Application to Include PD in the NBS

On the basis of the criteria set out in the new NBS National Policy Framework, the Australian Pompe Association took the initiative to submit an application to add IOPD to the NBS program. An initial application was submitted in 2018 but was not reviewed; it was then resubmitted in November 2018 to meet the timelines for a 2019 meeting and decision.

As part of its submission, the Australian Pompe Association calculated that the cost of first-tier dry blood spot testing for PD would be $8.78, amounting to approximately AUD$2.7 million each year, assuming an estimated total birth rate of 310,000 per annum and testing via established tandem mass spectrometry methods. However, recognized additional cost considerations included the need for each State/Territory to purchase a new mass spectrometer because current facilities are believed to be at capacity, funding for additional staff to manage this workload, the establishment and provision of genetic counseling services and costs incurred in second-tier confirmatory testing.

A key consideration of the application was its focus on IOPD, given that this has the most benefit to be gained by minimizing the diagnostic delay. It was hoped that, at the very least, this application would have been seen as a positive step forward and enable a pilot program to be implemented. A pilot program to evaluate first-tier testing has been developed but not implemented due to lack of funding and clinical resources. Such a program would help to define the prevalence of PD in Australia, enable evaluation of the optimal methodology for measuring GAA activity, and better inform costs and resourcing needs for NBS. In addition, it would aid in establishing post-testing diagnostic and confirmatory protocols and procedures for managing JOPD, LOPD, and patients with pseudodeficiency alleles that can lead to false positives.

After consideration by the Standing Committee on Screening, the application has not progressed to a more detailed review. The primary reasons for this being limited longitudinal evidence of survival improvements in treated IOPD patients as a result of identification via NBS and concerns regarding the negative impact of identifying patients at risk of developing LOPD. To a large extent, as has been discussed in this paper, literature providing answers to these concerns is becoming more readily available.

The rapid pace at which new data are emerging, coupled with the increase in uptake of NBS in developed countries like the USA, underscores the drive of the clinical, research and patient communities to provide earlier diagnosis and better outcomes for PD patients and their families. The Australian Pompe Association is encouraged by this and will seek to resubmit its nomination for adding IOPD to the Australian NBS program in the future.

### 4.3. Access to Current Therapies in Australia

Australia has an established and advanced program for the treatment of rare diseases, the Life Saving Drugs program (LSDP) was established in the mid-1990s and provides people with rare and life-threatening disease with access to medicines that are not listed on the Pharmaceutical Benefits Scheme. Currently 10 conditions are supported by the program, and of the treatments available alglucosidase alfa (Myozyme^®^) is subsidized for the treatment of IOPD, JOPD, and LOPD.

The LSDP requires that patients meet specific conditions to obtain access to treatment; including initial and ongoing eligibility criteria, and that they undergo annual reviews. Clear protocols are in place for diagnosing the disease and for its ongoing management, including starting and stopping treatment.

The decision by the Australian government to fund treatment through the LSDP is an example to all countries for programs to establish treatment for minority patient groups who face the challenge of living with a rare disease. Treatment is expensive. The only currently approved treatment is ERT and a recent review of the economic costs of PD has established that while available data demonstrate a high cost to patients and healthcare systems, there are substantial gaps in the literature [46]. It is hoped that as new treatment options became available, and competitive interest develops, production methodology will become more cost effective enabling the overall cost of ERT treatment to decline.

### 4.4. Potential Impact of Future Therapies

Prior to 2006, the only therapy available to patients with PD was palliative. ERT, the only currently available treatment for PD, has been very successful; it can extend the lifespan of babies born with IOPD and stabilize disease progression in patients with LOPD. However, it does not represent a cure. Research continues with many potential avenues including investigations into other therapies such as immune modulation, upregulation of receptor expression, second-generation recombinant ERT, chaperone therapy, substrate reduction therapy, and gene therapy [47]. Recent reviews provide up-to-date information of the available data for these potential new therapies [48,49].

Amongst these therapies, the prospect of gene therapy is of great interest. Currently several biotechnology companies are actively developing gene therapies for PD, while some therapies are still in preclinical development, other have entered early phase clinical trials [48]. Gene therapy has the potential to enable sustained enzyme supply after a single medical intervention; by enabling the patient to produce his or her own enzyme it will vastly change the way in which PD is managed.

As we enter the 2020s, for the first time in 56 years we have an opportunity to significantly reduce the suffering, distress and despair that a diagnosis of PD brings. NBS combined with the potential for gene therapy provides hope that in the not too distant future such patients will be able to say ‘*Yes I had PD as a baby, but I am fine now thanks to my early detection and treatment*’.

## 5. Conclusions

NBS has emerged over the past decade as an important contributor to more timely diagnosis and treatment of PD, particularly for babies with IOPD who would otherwise not survive and pass away with their true diagnosis undocumented. Early diagnosis and early access to treatment are pivotal to optimal clinical outcomes.

Australia is not alone in not yet having an NBS program for PD. Here, as in many other countries, the current scenario for patients with PD involves a lengthy diagnostic journey and belated commencement of treatment; in the case of IOPD often after considerable damage has already occurred. NBS facilitates earlier diagnosis and treatment access in a disease in which this timing is absolutely crucial. Identifying and treating IOPD earlier can make a difference between survival and death, between positive outcomes and severe disability. Existing NBS programs have demonstrated the ability to improve patients’ lives. Thus, despite its challenges, these positives greatly outweigh the negatives. While we have not had a positive outcome from our application, we hope that by taking the initiative to submit a proposal to include IOPD on the Australian NBS program it will encourage other groups elsewhere to be proactive in investigating and utilizing whatever systems are available in their countries to make similar applications.

The Pompe patient community, both in Australia and around the globe, is highly supportive of NBS [50]. Much headway has been made in ensuring that this patient voice is heard by medical specialists, scientific researchers and industry. Now it is time that this voice is also heard by the regulator to ensure equal and equitable access to NBS and the many benefits it can bring to the lives of PD patients and their families.

## Figures and Tables

**Table 1:** Australian survey data: Diagnostic delays are common in rare diseases.

Age Group	Results	Reference
**Adults**	Time to diagnosis: 1 year in 51.2% of cases ≥5 years in 30.0% of cases Number of doctors seen to get confirmed diagnosis: 1–2 in 33.7% of cases 3–5 in 37.4% of cases ≥6 in 28.8% of cases Number with at least one incorrect diagnosis: 45.9% of cases	Molster, 2016 [13]
**Children**	Time to diagnosis: 1 year in 59.8% of cases ≥3 years in 8.0% of cases Number of doctors seen to get confirmed diagnosis: 1–2 in 12.5% of cases 3–5 in 41.8% of cases ≥6 in 27.7% of cases Number with at least one incorrect diagnosis: 27.3% of cases	Zurynski, 2017 [12]
**Key Considerations**: Receiving a diagnosis of a rare disease is a life-changing event; delays in receiving a diagnosis are associated with anxiety, stress, symptomatic worsening, inappropriate use of resources and lack of access to appropriate support and care; Health professional education is needed to increase awareness of rare diseases and improve the diagnostic process; Resources, including access to multi-disciplinary care teams, are needed to support the requirements of people newly diagnosed with rare diseases.		

**Table 2:** Results from newborn screening (NBS) programs for Pompe disease (PD). Adapted from Bodamer 2017 [21].

Country and Region	Sample Size	Total Cases of IOPD	Total Cases of LOPD	Prevalence
Taiwan	473,738	9	19	1/16,919
Austria *	34,736	0	4	1/8684
Italy *	3403	0	0	-
Hungary *	40,024	7	2	1/4400
USA (State):				
Illinois *	166,463	2	9	1/15,133
Missouri	269,500	4	20	1/11,229
Washington *	154,544	0	5	1/31,000
New York	390,000	1	30	1/165,000

* Pilot studies.
